# Supplementary material for: Pocket similarity identifies selective estrogen receptor modulators as microtubule modulators at the taxane site
Source: Nat Commun. 2019 Mar 4;10:1033. doi: 10.1038/s41467-019-08965-w (PMC6399299; doi:10.1038/s41467-019-08965-w)
Supplement: Supplementary file 1 — Supplementary Information [file 41467_2019_8965_MOESM1_ESM.pdf]

Supplementary Information

Pocket Similarity Identifies Selective Estrogen Receptor Modulators as Microtubule Modulators at the Taxane Site

Lo *et al.*

Table of Contents

Supplementary Figure 1: Microtubule effects of overnight treatment with 50 μM of each SERM.

Supplementary Figure 2: Representative images of SERM-induced microtubule defects.

Supplementary Figure 3: Stabilization of microtubules by a short treatment with 50 μM of raloxifene.

Supplementary Figure 4: Negative stain electron microscopy of microtubules incubated with SERMs.

Supplementary Figure 5: SiR-tubulin displacement by SERMs in vivo.

Supplementary Figure 6: Quantification of cell growth during all treatments for EC_{50} determination.

Supplementary Figure 7: Quantification of cell death during all treatments for EC_{50} determination.

Supplementary Figure 8: Live imaging of raloxifene-treated cells reveals mitotic defects.

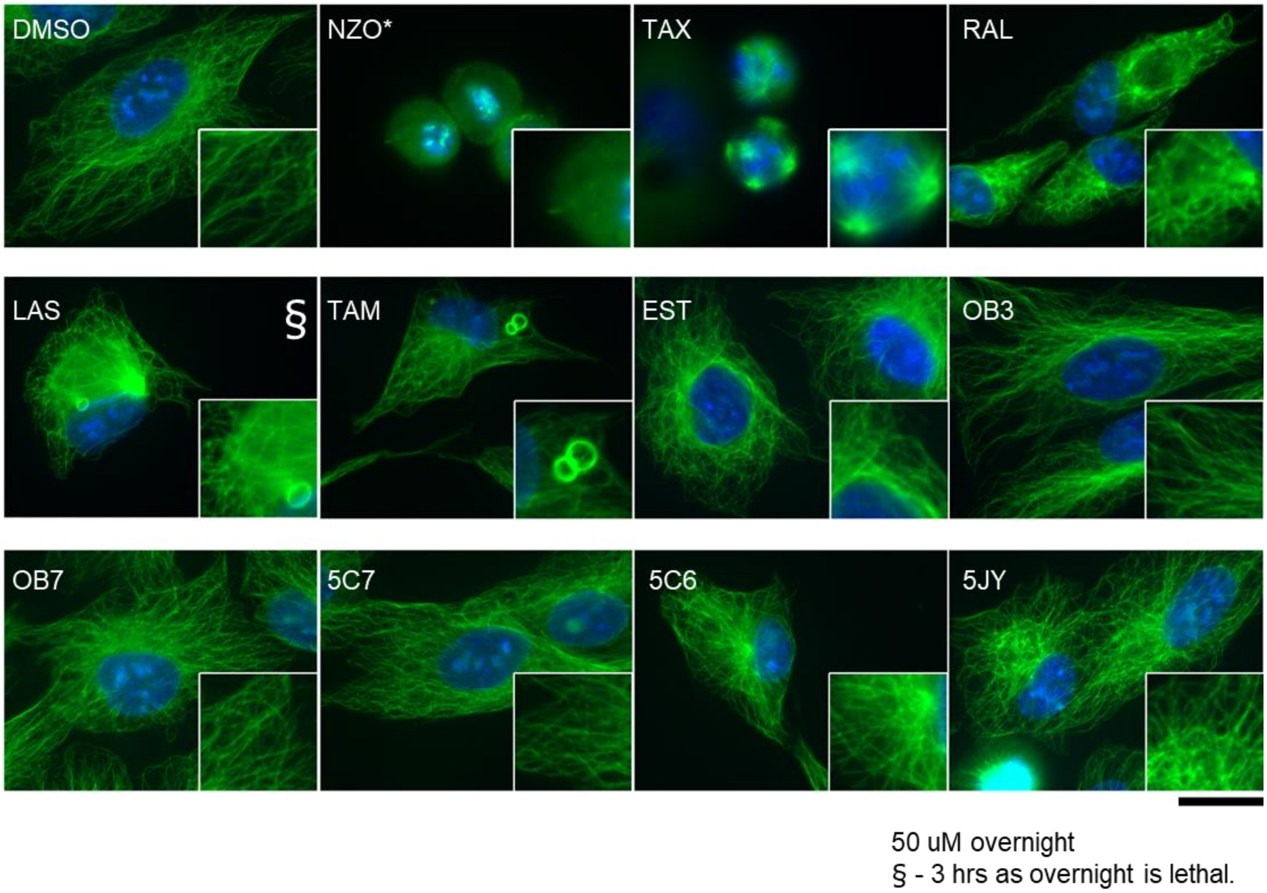

Supplementary Figure 1: Microtubule effects of overnight treatment with 50 µM of SERMs. hTERT-RPE1 cells were treated with 50 µM of each SERMs. The asterisk (*) indicates 500 nM. § indicates 3 hours rather than overnight treatment, as overnight treatment is mostly lethal. Scale bar 20 µm. Inset is approximately 200%.

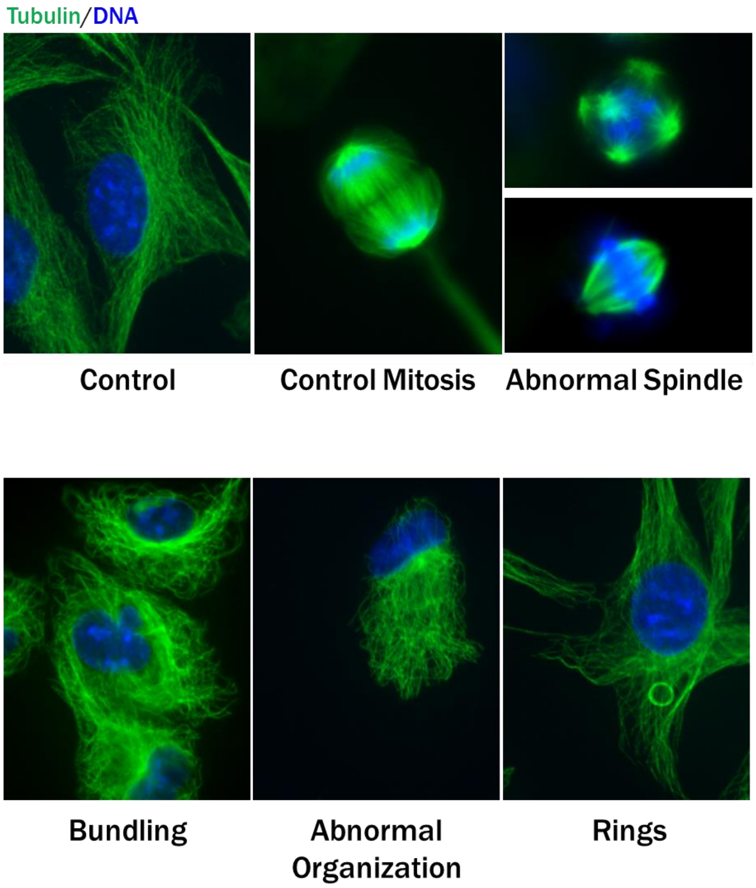

Supplementary Figure 2: Representative images of SERM-induced microtubule defects.

Treated RPE-1 cells stained for alpha-tubulin (green), and DNA (blue). Representative images of scored microtubule morphologies in Supplementary Data 4.

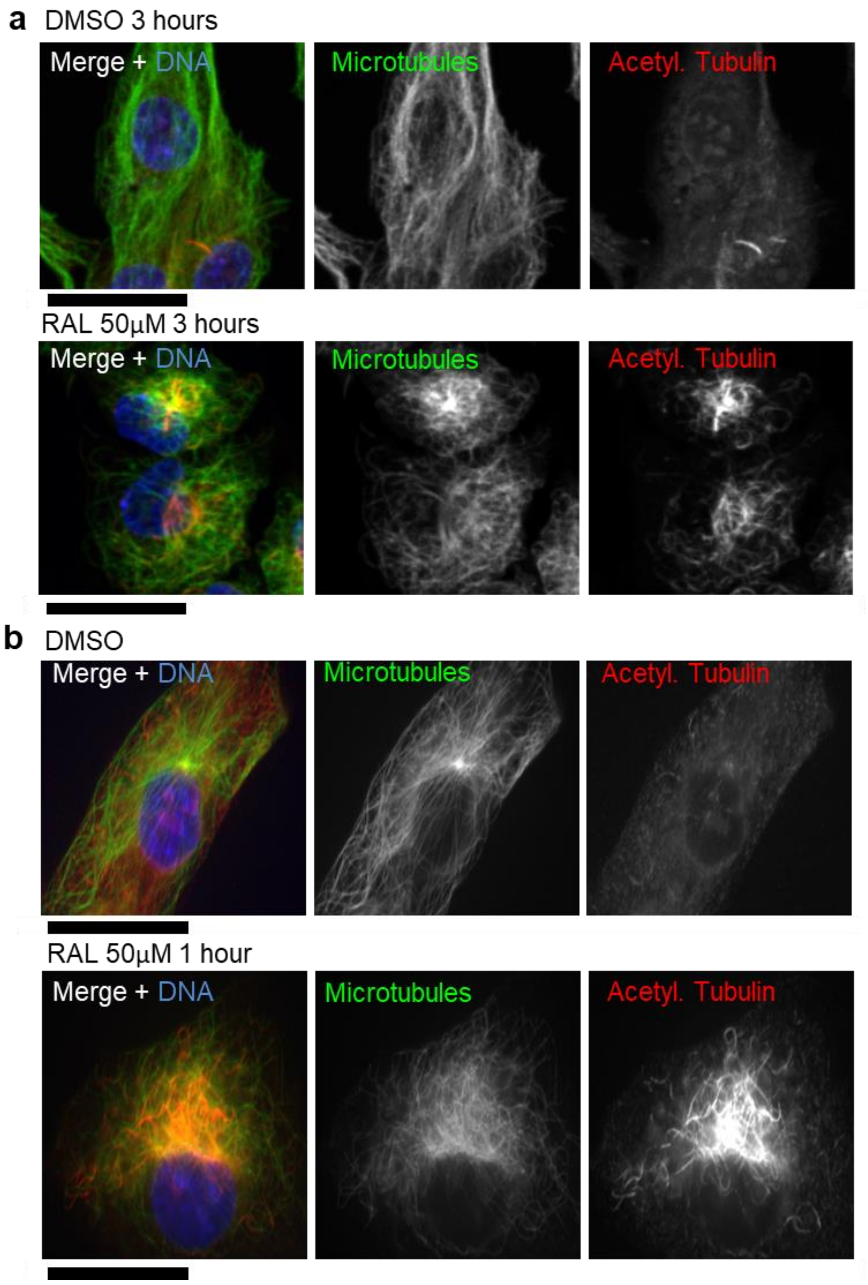

Supplementary Figure 3: Stabilization of short treatment with 50 µM of raloxifene. hTERT-RPE1 cells were treated as indicated, and immunostained for alpha-tubulin and acetylated tubulin. Scale bar 20 µm. (a) Contrasts the extent of tubulin acetylation after 3 hours of treatment with raloxifene or vehicle. (b) Contrasts the extent of tubulin acetylation after 1 hour of treatment with raloxifene or vehicle.

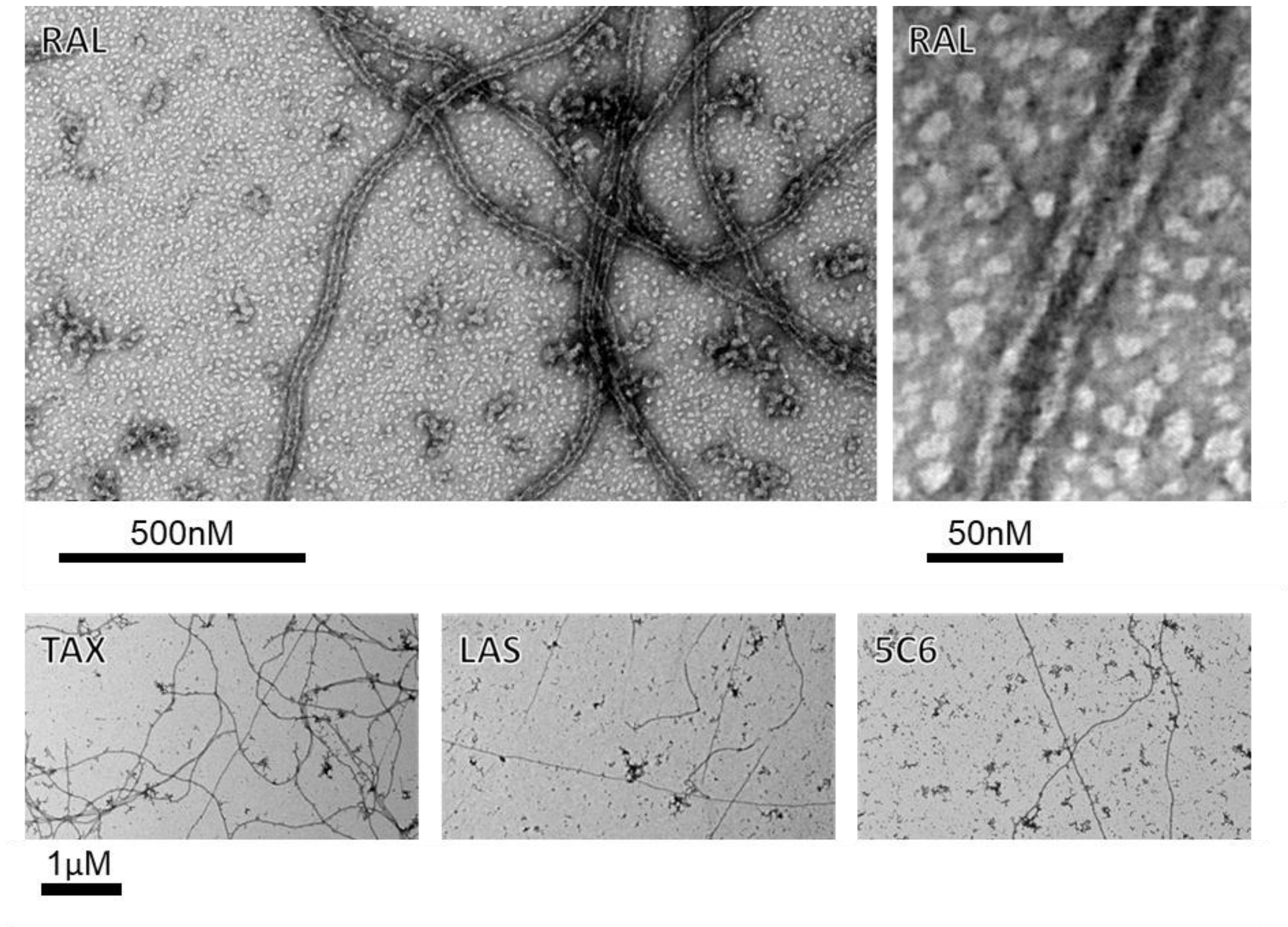

Supplementary Figure 4: Negative stain electron microscopy of microtubules incubated with SERMs. Polymerized *in vitro* microtubules in the presence of indicated drugs (as in Figure 4c) imaged using negative stain electron microscopy. Scale bars and drugs used as indicated.

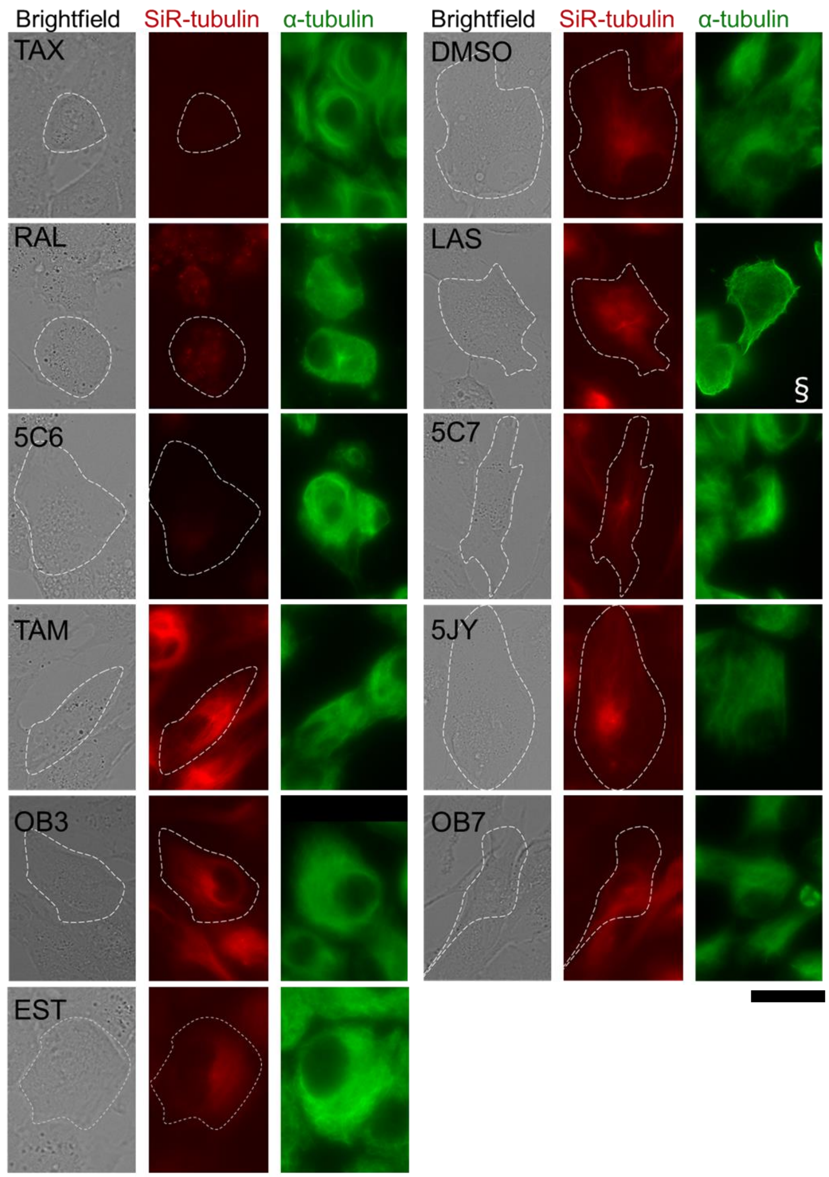

Supplementary Figure 5: SiR-tubulin displacement by SERMs *in vivo.* hTERT-RPE1 cells were treated with SiR-tubulin, verapamil and each SERM in excess for 3 hours. Indicated is the brightfield signal, SiR-tubulin signal with the cell outlined. At the conclusion of the experiment, cells were fixed with methanol and immunostained against alpha-tubulin (show as a sum projection of 10 z-slices). Note all cells regardless of the SiR-tubulin staining have intact microtubules network and methanol fixation may result in slight cell morphology changes. § indicates a cell from the same treatment, but not the original cell due to its death. Scale bar 35 μm.

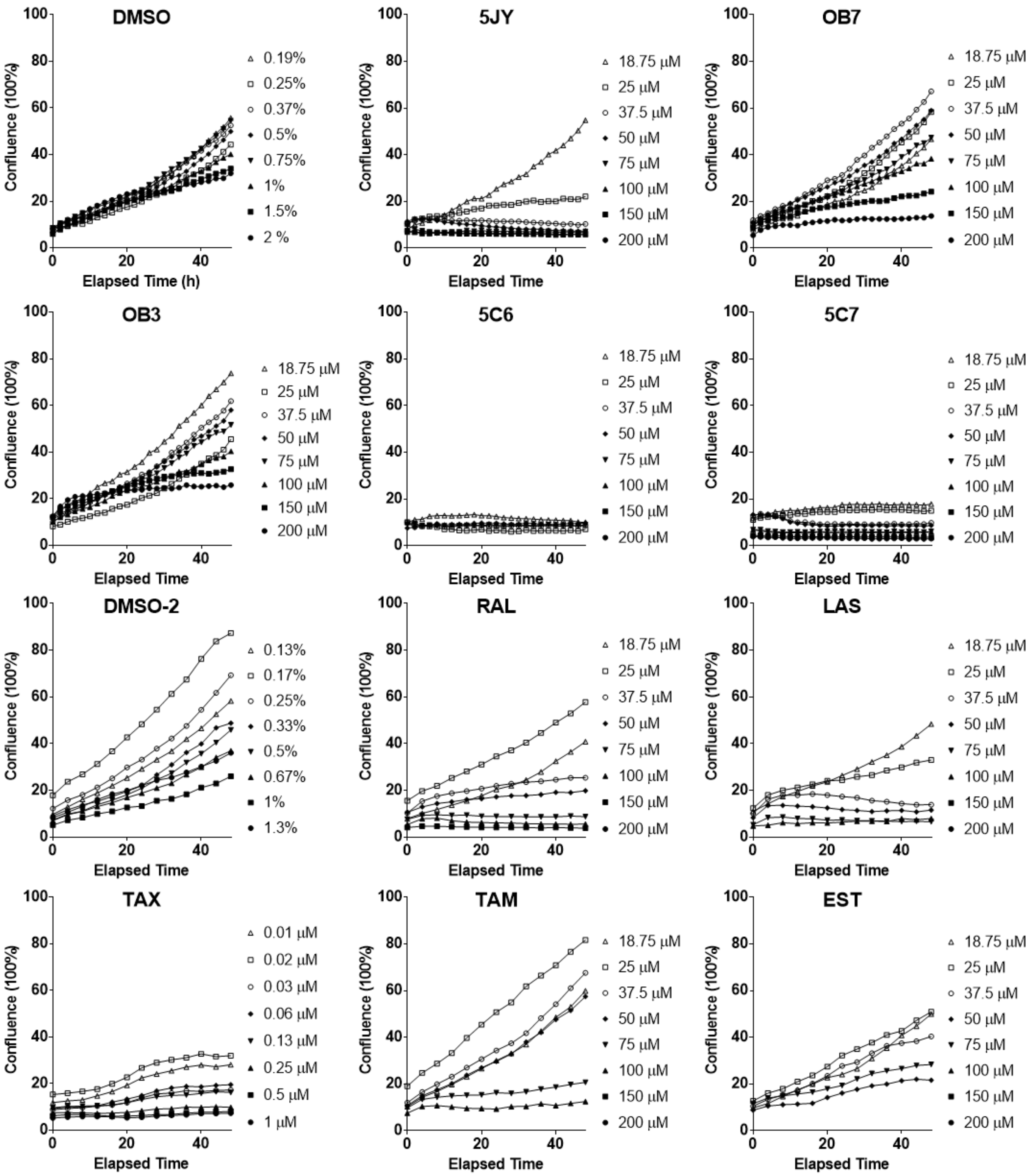

Supplementary Figure 6: Quantification of cell growth during all treatments for EC_{50} determination. hTERT-RPE1 cells were treated with SERMs for 30 minutes, followed by live imaging over two days in the presence of YOYO-3.

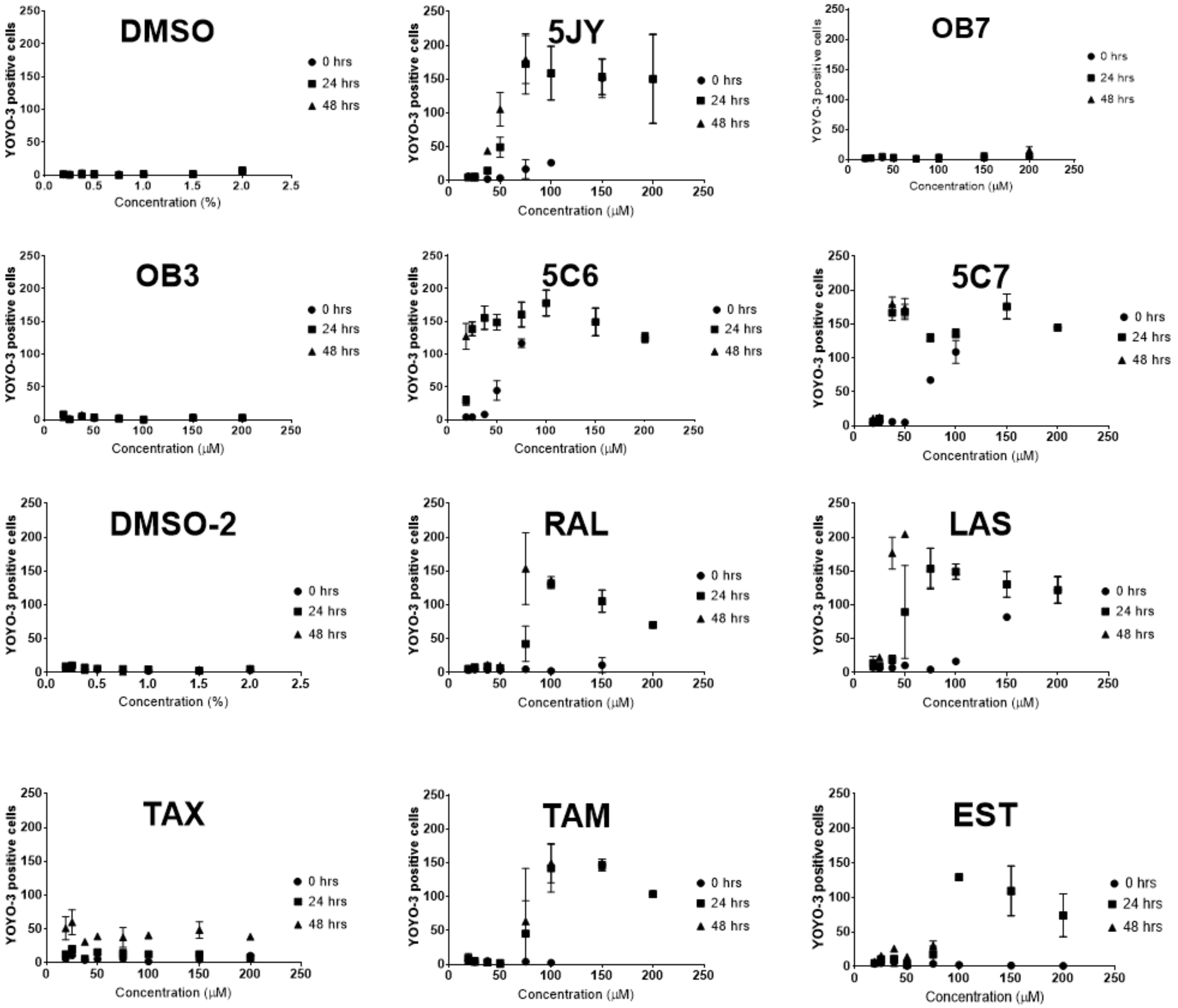

Supplementary Figure 7: Quantification of cell death during all treatments for EC_{50} determination. hTERT-RPE1 cells were treated with SERMs for 30 minutes, followed by live imaging over two days in the presence of YOYO-3. Note that the x-axis is the compound test concentration (μM) and the y-axis is the number of YOYO-3 positive cells counted. Error bars represent standard deviation.

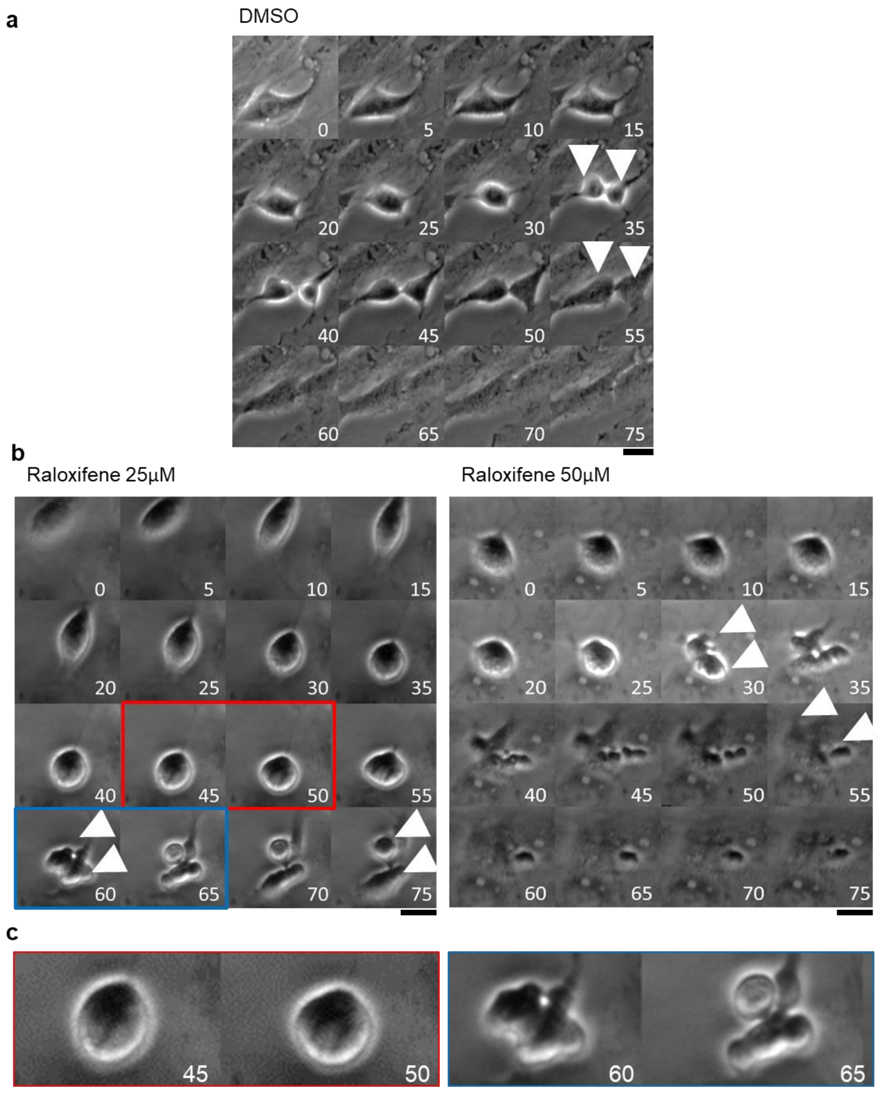

Supplementary Figure 8: Live imaging of raloxifene-treated cells reveals mitotic defects. hTERT-RPE1 cells were treated with DMSO or raloxifene and imaged using phase-contrast for several hours. Representative mitotic events during the imaging period are shown, with minutes indicated. Arrowheads indicate events when cells first separate into daughters, and one (or both) cells returning to the monolayer. (a) DMSO treated control cells undergoing a mitotic division. (b) Raloxifene treated cells at 25 µM and 50 µM undergoing a mitotic division. Note the blebbing following anaphase. (c) Detailed view of the mitotic events indicated from (b). Note the transition from metaphase to anaphase between 45 and 50 minutes, and the following mitotic failure at 60 and 65 minutes. All scale bars are estimated 13 µm.
