# Supplementary material for: Pocket similarity identifies selective estrogen receptor modulators as microtubule modulators at the taxane site
Source: Nat Commun. 2019 Mar 4;10:1033. doi: 10.1038/s41467-019-08965-w (PMC6399299; doi:10.1038/s41467-019-08965-w)
Supplement: Supplementary file 3 — Description of Additional Supplementary Files [file 41467_2019_8965_MOESM3_ESM.pdf]

Supplementary Movie Legends:

Title: Supplementary Movie 1
Description: Growth of DMSO-treated (0.5%) hTERT-RPE1 cells, with YOYO-3.

Title: Supplementary Movie 2
Description: Growth of RAL-treated (50 µM) hTERT-RPE1 cells, with YOYO-3.

Title: Supplementary Movie 3
Description: Growth of LAS-treated (50 µM) hTERT-RPE1 cells, with YOYO-3.

Title: Supplementary Movie 4
Description: Growth of 5C6-treated (50 µM) hTERT-RPE1 cells, with YOYO-3.

Title: Supplementary Movie 5
Description: Growth of 5C7-treated (50 µM) hTERT-RPE1 cells, with YOYO-3.

Title: Supplementary Movie 6
Description: Growth of OB7-treated (50 µM) hTERT-RPE1 cells, with YOYO-3.

Title: Supplementary Movie 7
Description: Growth of all treatments side-by-side (from Supplementary Movie 1-7).

Supplementary Data Legends:

Title: Supplementary Data 1
Description: PocketFeature Scores for compounds in the broad in silico similarity screen.

Title: Supplementary Data 2
Description: Predicted affinities for predicted taxane binding compounds.

Title: Supplementary Data 3
Description: PocketFeature Scores for compounds in the targeted estrogen receptor screen.

Title: Supplementary Data 4
Description: Quantification of immunofluorescence microtubules effects observed.

Title: Supplementary Data 5
Description: Compounds used in the manuscript and their sources.

Title: Supplementary Data 6
Description: NCI60 cell line SERM treatment based on the ER status.
